# Insights into refractory chronic inflammatory demyelinating polyneuropathy: a comprehensive real-world study

**DOI:** 10.3389/fneur.2024.1326874

**Published:** 2024-01-31

**Authors:** Yongsheng Zheng, Jianian Hu, Chong Sun, Kai Qiao, Yanyin Zhao, Bingyou Liu, Jian Sun, Jianying Xi, Sushan Luo, Jiahong Lu, Chongbo Zhao, Jie Lin

**Affiliations:** ^1^Department of Neurology, Huashan Hospital of Fudan University, Shanghai, China; ^2^National Center for Neurological Disorders (NCND), Shanghai, China; ^3^Huashan Rare Disease Center, Huashan Hospital Fudan University, Shanghai, China

**Keywords:** chronic inflammatory demyelinating polyneuropathy, refractory, disease course form, prognosis, electrophysiological characteristics

## Abstract

**Background:**

Refractory chronic inflammatory demyelinating polyneuropathy (CIDP) is a challenging subset of CIDP. It does not respond well to immune therapy and causes substantial disability. A comprehensive understanding of its clinical profile, electrophysiological characteristics and potential risk factors associated with refractoriness remains to be further elucidated.

**Methods:**

Data in this cross-sectional study was collected and reviewed from the Huashan Peripheral Neuropathy Database (HSPN). Included patients were categorized into refractory CIDP and non-refractory CIDP groups based on treatment response. The clinical and electrophysiological characteristics were compared between refractory and non-refractory CIDP groups. Potential risk factors associated with refractory CIDP were explored with a multivariate logistic regression model.

**Results:**

Fifty-eight patients with CIDP were included. Four disease course patterns of refractory CIDP are described: a relapsing–remitting form, a stable form, a secondary progressive form and a primary progressive form. Compared to non-refractory CIDP patients, refractory CIDP exhibited a longer disease duration (48.96 ± 33.72 vs. 28.33 ± 13.72 months, *p* = 0.038) and worse functional impairment (MRC sum score, 46.08 ± 12.69 vs. 52.81 ± 7.34, *p* = 0.018; mRS, 2.76 ± 0.93 vs. 2.33 ± 0.99, *p* = 0.082; INCAT, 3.68 ± 1.76 vs. 3.03 ± 2.28, *p* = 0.056, respectively). Electrophysiological studies further revealed greater axonal impairment (4.15 ± 2.0 vs. 5.94 ± 2.77 mv, *p* = 0.011, ulnar CMAP) and more severe demyelination (5.56 ± 2.86 vs. 4.18 ± 3.71 ms, *p* = 0.008, ulnar distal latency, 7.94 ± 5.62 vs. 6.52 ± 6.64 ms, *p* = 0.035, median distal latency; 30.21 ± 12.59 vs. 37.48 ± 12.44 m/s, *p* = 0.035, median conduction velocity; 58.66 ± 25.73 vs. 42.30 ± 13.77 ms, *p* = 0.033, median F-wave latency), compared to non-refractory CIDP. Disease duration was shown to be an independent risk factor for refractory CIDP (*p* < 0.05, 95%CI [0.007, 0.076]).

**Conclusion:**

This study provided a comprehensive description of refractory CIDP, addressing its clinical features, classification of clinical course, electrophysiological characteristics, and prognostic factors, effectively elucidating its various aspects. These findings contribute to a better understanding of this challenging subset of CIDP and might be informative for management and treatment strategies.

## Introduction

CIDP is an immune-mediated radiculoneuropathy, characterized by proximal and distal limb weakness and numbness, and absent or reduced tendon reflexes at four limbs (1, 2). Although most of the patients respond well to first-line immune treatment including immunoglobulin therapy [intravenous (IVIg) or subcutaneous Ig], corticosteroids, or therapeutic plasma exchange (TPE), 20–30% of CIDP patients do not adequately respond to these therapies, and around 6 to 15% of patients remain refractory to all treatment (3–5).

The existing literature lacks a comprehensive description of the clinical features, electrophysiological findings and overall prognosis of this subset of CIDP patients (6–9). Moreover, risk factors for patients being refractory to treatment are not completely clear. Traditionally, CIDP variants (such as multifocal CIDP), insidious onset, progressive course, central nervous system involvement, and irreversible axonal degeneration have been considered as factors contributing to refractoriness in CIDP (6, 10, 11). Previous studies on refractory CIDP had included patients with chronic immune sensory polyradiculopathy (CISP) and/or IgG4 antibody related autoimmune nodopathy. Recently studies have revealed that autoimmune nodopathy, formerly considered as a subset of CIDP and accounting for approximately 10% to 20% of the total cases, clinically presents as refractory CIDP (12, 13). In 2021 European Academy of Neurology/Peripheral Nerve Society (EAN/ PNS) guideline (14), autoimmune nodopathy and CISP were not classified as CIDP. Hence, risk factors as well as a complete clinical profile for refractory CIDP under the new guideline are completely unknown.

In this study, we strictly applied the 2021 EAN/PNS clinical criteria for CIDP to a cohort of neuropathy patients sourced from a national rare disease center database. Our primary objectives were to describe the clinical presentation, disease course form, as well as electrophysiological characteristics of refractory CIDP. Additionally, we aimed to investigate potential risk factors associated with refractory CIDP. Through this research, we aimed to expand our understanding of this challenging subset of CIDP and contribute to improving management and treatment strategies.

## Methods

### Huashan peripheral neuropathy database

The data of present study was from the HSPN database of the National Rare Disease Center, Huashan Hospital, Shanghai, China. In the HSPN database, patients with “suspected CIDP” was defined as: (1) subjects that fulfilled the required clinical features of CIDP including the typical form, or of any clinical variant; (2) subjects were required to demonstrate demyelination features based on electrophysiological evaluation, although strict adherence to the criteria outlined in the EFNS/PNS Guidelines (15) (prior to July 2021) or the updated EAN/PNS Guidelines (after July 2021) was not mandatory (14); and (3) other etiologies that could cause CIDP were excluded at the time of enrollment into HSPN database. The inclusion of all such clinical cases may, therefore, obviously lead to erroneously high sensitivity calculation for the disease overall. The ethical approval was obtained from the Ethics Committee of Huashan hospital, Fudan university and have been performed in accordance with the ethical standards laid down in the 1964 Declaration of Helsinki and its later amendments.

### Study population

Data from patients with “suspected CIDP” was retrospectively retrieved from the HSPN database. All patients with “suspected CIDP” that had complete medical data underwent a detailed clinical history including time of onset, disease duration, distribution and progression of signs and symptoms including weakness, sensory symptoms, gait disturbance, ataxia, pain, tremor, cranial nerve involvement, autonomic dysfunction and treatment response. The results of examinations, including cerebrospinal fluid (CSF) analysis, nerve ultrasound or brachial/ lumbosacral plexus MR examination, nerve conduction studies performed at baseline or during the course of the disease, somatosensory evoked potentials (SSEP) and sural nerve biopsy, were reported when available. Albuminocytological dissociation in the CSF analysis was defined as an increased protein level (>0.60 g/L) in the absence of elevated white cell count (<8 cells/μL) (16).

Neurological functional impairment and subjective assessment before and after each treatment were carefully reviewed. In our study, patients were routinely followed up every 3–6 months. Response to treatment was defined as an improvement that was objectively confirmed by the following clinical scales: (1) an increase in at least 4 points on the Medical Research Council sum score (MRC sum score, range 0–60); or (2) a decrease at least 1 point on the Inflammatory Neuropathy Cause and Treatment disability score (INCAT, range 0–10); or (3) a decrease at least 1 point on modified Rankin Scale (mRS, range 0–5).

Two senior neuromuscular specialists carefully reviewed patients’ medical history and nerve conduction studies. Firstly, patients with “suspected CIDP” met the 2021 EAN/PNS Guidelines as well as with a disease duration more than 6 months were included in this study. In this included population, patients with CIDP were further divided into two groups, the refractory CIDP group and the non-refractory CIDP group. Refractory CIDP was defined as following (17, 18): (1) no response to at least two of three first-line treatments (corticosteroids, IVIg, or TPE) or relapse during drug tapering off; or (2) dependence on at least two of three first-line treatments simultaneously for maintain treatment; or (3) no response to at least one of three first-line treatments combined with one of immunosuppressive drugs (rituximab, azathioprine, mycophenolate mofetil, methotrexate, fingolimod or cyclophosphamide). CIDP patients not fulfilling this definition were considered as non-refractory CIDP and were included for comparison.

Furthermore, within the refractory CIDP group, we specifically focused on patients who had a clinical follow-up duration of over 1 year and had a minimum of more than three follow-up visits throughout their disease course. Through this stringent filtering process, we identified a subgroup of patients for whom we thoroughly reviewed and described the different disease course patterns. We define the relapsing–remitting form as a condition where patients experience symptomatic improvement with the initiation of treatment, followed by a subsequent exacerbation of symptoms upon cessatin of therapy. This pattern of response and deterioration periodically occurs, leading to fluctuating clinical symptoms over time. The criteria for defining improvement and exacerbation are based on changes in clinical scores, as detailed previously. Further, we delineate a stable course as one where the patient’s condition neither improves nor deteriorates, maintaining a consistent plateau post-treatment. In contrast, a progressive course is defined by a continuous decline in clinical symptoms despite therapeutic interventions. This includes the ‘primary progressive form,’ where deterioration is persistent from onset, and the ‘secondary progressive form,’ where clinical symptoms exacerbate following an initial phase of improvement.

At the time of our study inclusion, patients with an alternative diagnosis for the neuropathy or patients with concomitant hematological disorders associated with monoclonal gammopathy were excluded. Patients with antibodies against nodal/paranodal cell adhesion molecules (contactin-1 [CNTN1], neurofascin-155 [NF155], contactin-associated protein 1 [Caspr1], and neurofascin isoforms NF140/186) and patients with CISP were excluded. We employed a cell-based assay method for the initial screening of node/paranodal antibodies, followed by the rat teased fiber immunofluorescence assay for confirmation, as detailed in our previous publications (19, 20). Additionally, patients with central combined with peripheral demyelination (CCPD) were also excluded.

### Statistical analysis

Categorical variables are described using frequencies and percentages, while continuous variables are described using mean and standard deviation (SD). Comparisons between the refractory CIDP and non-refractory CIDP groups were performed using Chi-square test or Fisher’s exact test, t-test or Wilcoxon rank sum test, as appropriate. To assess the relationship between the status of being refractory and various clinical indicators, we initiated our analysis with univariate analyses, incorporating those variables with *p*-values less than 0.05 into the binary regression analysis. In order to evaluate multicollinearity, we calculated the Variance Inflation Factor (VIF) for each variable. A VIF value exceeding 5 is indicative of the presence of multicollinearity. Ultimately, we performed a logistic regression analysis, excluding variables with VIF greater than 5. During this process, we handled missing values by directly dropping the missing values. We calculated the coefficients, standard errors, 95% confidence interval (95% CI) and *p*-value of the independent variables. Analyses were performed and figures were generated with the R software (R version 4.2.2) and Python 3.10. All tests are two-tailed, and the significance level is set to 0.05.

## Results

### Study population selection

**Figure 1:**
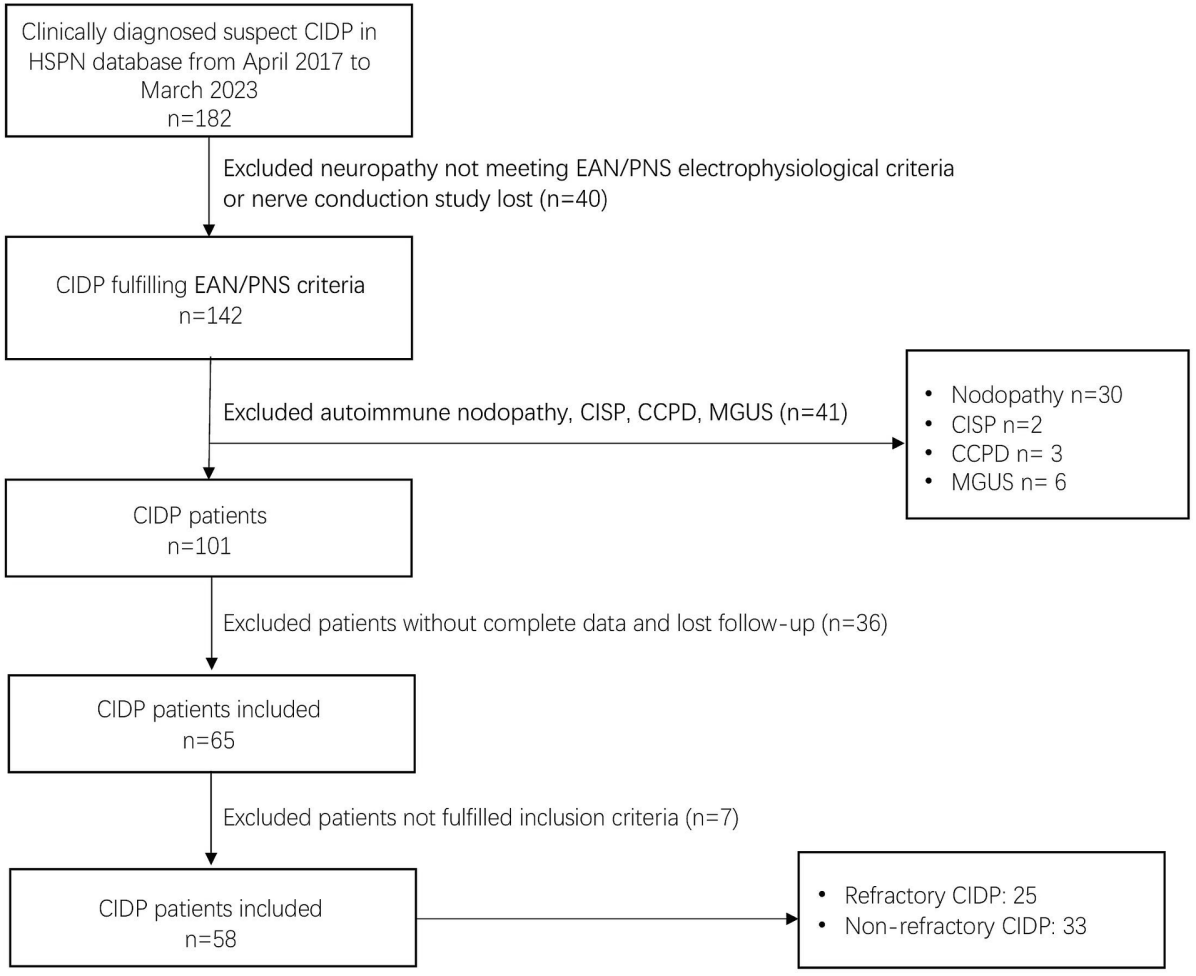
Flowchart of patient cohort enrolment and exclusion.

Among the 182 patients labeled as “suspected CIDP” in the HSPN database from April 2017 to March 2023, 142 patients were included, all of whom had available nerve conduction study data and met the EAN/PNS electrophysiological criteria. Of these confirmed CIDP population, 41 patients were excluded, including 30 patients with autoimmune nodopathy (18 with anti-NF155, 8 with anti-NF186, 3 with anti-CNTN1, and 2 with anti-Caspr1), 2 patients with CISP, 3 patients with CCPD and 6 patients with concomitant hematological disorders associated with monoclonal gammopathy. Among the 101 patients with CIDP, 36 patients were further excluded because of incomplete clinical data or loss to follow-up, or not fulfilling study inclusion criteria. Furthermore, 7 patients were excluded because of not fulfilling our inclusion criteria. Fifty-eight patients were included in the final study population (Figure 1).

### Clinical characteristics of refractory CIDP

In our study, the demographic and clinical features of 25 refractory CIDP patients at their initial consultation in our hospital were summarized and compared with those of patients with non-refractory CIDP. This comparison includes both patients who had not previously received any treatment and those who had undergone treatment at other institutions (Table 1). There were 20 males (80.0%) in the refractory CIDP group, with a mean age at symptom onset of 44.15 ± 18.29 years. According to the 2021 EAN/PNS guideline, 14 (56.0%) patients were typical CIDP and 11 patients were CIDP variants (7 distal CIDP, 1 multifocal CIDP, 1 focal CIDP, 1 motor CIDP and 1 sensory CIDP). Most of the refractory CIDP patients (72.0%) had a chronic onset. The refractory CIDP group had a disease duration of 48.96 ± 33.72 months, significantly longer than that in non-refractory CIDP (28.33 ± 13.72 months, *p* = 0.038). Refractory CIDP patients exhibited a more severe functional impairment compared with non-refractory CIDP patients (MRC sum score, 46.08 ± 12.69 vs. 52.81 ± 7.34, *p* = 0.018; mRS, 2.76 ± 0.93 vs. 2.33 ± 0.99, *p* = 0.082; INCAT, 3.68 ± 1.76 vs. 3.03 ± 2.28, *p* = 0.056, respectively). There was no difference in treatment response to IVIg between these two groups. However, non-refractory CIDP patients had a better response to glucocorticoid and TPE (Table 1). Other demographic and clinical features did not demonstrate statistically significant differences. In our analysis, we specifically examined the prevalence of comorbidities such as diabetes and kidney disease, which are known to contribute to peripheral neuropathy. Our data indicated no statistically significant difference in the prevalence of these comorbidities between refractory and non-refractory CIDP patients, as detailed in the table provided (Supplementary Table S1).

**Table 1:** Clinical and laboratory characteristics of refractory CIDP patients.

	Refractory (*n* = 25)	Non-refractory (*n* = 33)	*p*-value
Gender (M: F)	20:5	18:15	0.082
Age onset (years)	44.15 ± 18.29	42.65 ± 15.12	0.495
Disease duration (months)	48.96 ± 33.72	28.33 ± 13.72	**0.038**
Follow-up duration (months)	24.24 ± 17.9	16.48 ± 10.49	0.061
Diagnosis			
CIDP	23, 92.0%	28, 84.85%	0.687
Possible CIDP	2, 8.0%	5, 15.15%	/
Variant classification			
Typical CIDP	14, 56.0%	18, 54.55%	1.0
CIDP variants	11, 44.0%	15, 45.45%	/
Distal	7, 28.0%	10, 30.3%	/
Multifocal and focal	2, 8.0%	3, 9.1%	/
Motor/Motor dominant	1, 4.0%	2, 6.1%	/
Sensory/Sensory-dominant	1, 4.0%	0, 0.0%	/
Onset			
Acute/subacute	7, 28.0%	7, 21.21%	0.773
Chronic	18, 72.0%	26, 78.79%	/
Symptoms at inclusion			
Weakness	24, 96.0%	30, 90.91%	0.627
Numbness	24, 96.0%	31, 93.94%	1.00
Symmetry	23, 92.0%	29, 87.88%	0.690
Pain	2, 8.0%	4, 12.12%	0.690
Gait disturbance	14, 56.0%	15, 45.45%	0.596
Cranial nerve involvement	3, 12.0%	3, 9.09%	1.00
Physical examination at inclusion			
Tremor	7, 28.0%	7, 21.21%	0.773
Vibration decrease/loss	14, 56.0%	13, 39.39%	0.332
Pinprick decrease/loss	17, 68.0%	20, 60.61%	0.761
Rombergs’ sign	11, 50.0%	10, 35.71%	0.467
Clinical scale at inclusion			
mRS	2.76 ± 0.93	2.33 ± 0.99	0.082
INCAT	3.68 ± 1.76	3.03 ± 2.28	0.056
MRC	46.08 ± 12.69	52.81 ± 7.34	**0.018**
CSF analysis			
Protein level (mg/dl)	1708.17 ± 1475.40	1770.09 ± 1386.37	0.895
Albuminocytological dissociationn	22, 91.67%	24, 75.0%	0.162
Nerve imaging	8, 72.73%	12, 70.59%	1.00
Overall response to first-line treatment			
glucocorticoid	9, 39.13%	25, 92.59%	**0.000**
IVIg	11, 64.71%	18, 90.0%	0.109
TPE	7, 46.67%	9, 100%	**0.010**

F, female; INCAT, Inflammatory Neuropathy Cause and Treatment Disability Score; M, male; MRC, Medical Research Council Sum Score; mRS, modified Rankin scale; TPE, therapeutic plasma exchange.

### Patterns of clinical course in refractory CIDP

**Figure 2:**
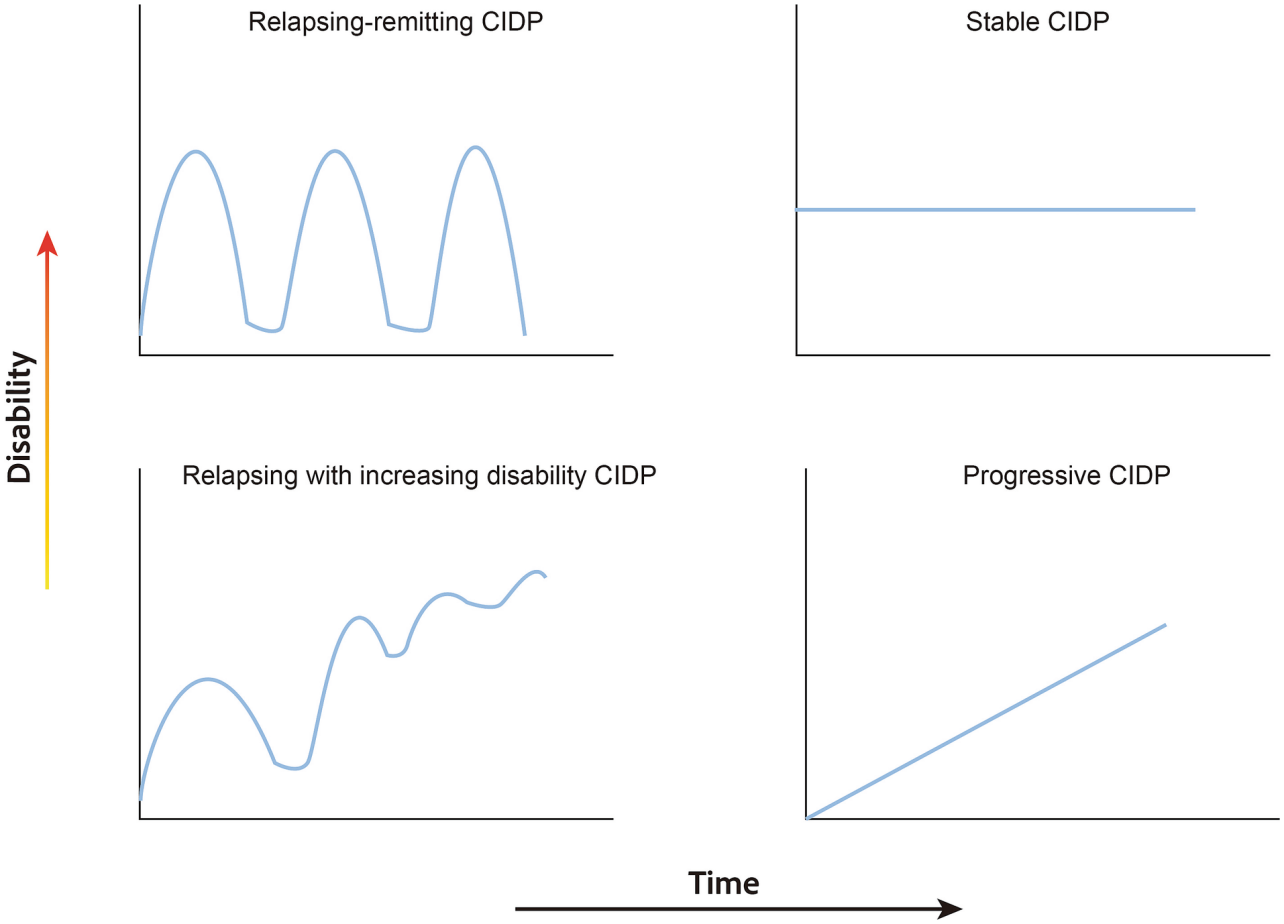
Schematic diagram of clinical course pattern of refractory CIDP.

Four patterns of clinical course in 21 refractory CIDP patients were summarized: relapsing–remitting form (9/21, 42.86%) (Supplementary Figure S1), stable form (4/21, 19.05%) (Supplementary Figure S2), primary progressive form (3/21, 14.29%) (Supplementary Figure S3), secondary progressive form (5/21, 23.81%) (Supplementary Figure S4). Schematic diagrams representing these four classifications are shown in Figure 2.

### Electrophysiological characteristics of refractory CIDP

Electrophysiological study were performed at patients’ initial consultation in our hospital. Nerve conduction characteristics of refractory CIDP patients were summarized and compared with patients with non-refractory CIDP in Table 2. In motor nerve studies, the refractory CIDP group showed significantly a lower ulnar compound muscle action potential (CMAP) (4.15 ± 2.0 vs. 5.94 ± 2.77 mv, *p* = 0.011), longer ulnar and median distal latency (5.56 ± 2.86 vs. 4.18 ± 3.71 ms, *p* = 0.008; 7.94 ± 5.62 vs. 6.52 ± 6.64 ms, *p* = 0.035, respectively), and a decreased median conduction velocity and a longer F-wave latency (30.21 ± 12.59 vs. 37.48 ± 12.44 m/s, *p* = 0.035, 58.66 ± 25.73 vs. 42.30 ± 13.77 ms, *p* = 0.033) compared to the non-refractory group. In the sensory nerve conduction study, refractory CIDP had a more decreased conduction velocity on the ulnar nerve compared to the non-refractory group (41.91 ± 9.14 vs. 49.21 ± 10.57, *p* = 0.037). No other significant statistical differences were found in other parameters and nerves.

**Table 2:** Electrophysiological characteristics of refractory CIDP patients.

Parameter	Refractory group	Non-refractory group	*p*-value
Motor nerve conduction study			
Ulnar nerve	*N* = 25	*N* = 33	
DML (ms)	5.56 ± 2.86	4.18 ± 3.71	0.008
CMAP (mv)	4.15 ± 2.00	5.94 ± 2.77	0.011
CV (m/s)	34.89 ± 15.30	37.34 ± 13.27	0.534
F-wave (ms)	54.56 ± 26.96	43.77 ± 13.58	0.131
Median nerve	*N* = 25	*N* = 33	
DML (ms)	7.94 ± 5.62	6.52 ± 6.64	0.035
CMAP (mv)	4.61 ± 2.70	5.24 ± 2.72	0.392
CV (m/s)	30.21 ± 12.59	37.48 ± 12.44	0.035
F-wave (ms)	58.66 ± 25.73	42.30 ± 13.77	0.033
Tibial nerve	*N* = 25	*N* = 33	
DML (ms)	9.55 ± 8.15	6.86 ± 4.81	0.254
CMAP (mv)	4.42 ± 4.88	4.78 ± 3.98	0.505
CV (m/s)	32.41 ± 10.66	32.25 ± 9.08	0.762
F-wave (ms)	70.34 ± 23.69	75.92 ± 14.94	0.460
Peroneal nerve	*N* = 23	*N* = 31	
DML (ms)	8.85 ± 6.06	7.21 ± 4.95	0.359
CMAP (mv)	2.48 ± 1.76	2.53 ± 1.84	0.926
CV (m/s)	31.69 ± 11.67	31.71 ± 8.54	0.995
F-wave (ms)	60.64 ± 20.25	66.04 ± 14.78	0.471
Sensory nerve conduction study			
Ulnar nerve	*N* = 24	*N* = 32	
SNAP (uv)	3.76 ± 2.90	5.48 ± 4.28	0.259
CV (m/s)	41.91 ± 9.14	49.21 ± 10.57	0.037
Median nerve	*N* = 23	*N* = 33	
SNAP (uv)	6.01 ± 6.14	9.85 ± 8.67	0.082
CV (m/s)	44.67 ± 10.87	49.79 ± 10.98	0.155
Sural nerve	*N* = 24	*N* = 31	
SNAP (uv)	9.72 ± 6.83	14.31 ± 12.38	0.371
CV (m/s)	47.62 ± 8.47	46.97 ± 9.00	0.936
NP in nerves			
Ulnar_nerve (motor)	1	0	0.888
Median_nerve (motor)	0	0	/
Tibial_nerve (motor)	4	4	0.968
Peroneal_nerve (motor)	4	7	0.870
Ulnar_nerve (sensory)	10	6	0.123
Median_nerve (sensory)	9	6	0.219
Sural_nerve (sensory)	7	6	0.569

CMAP, compound muscle action potential; CV, conduction velocity; DML, distal motor latency.

### Prognostic factors for evolving to refractory CIDP

For multivariate logistic regression analyses, the independent variables include disease duration, MRC sum score, ulnar nerve CMAP, median motor nerve distal latency and median motor nerve conduction velocity. In the assessment of the impact of various independent variables on potential risks of becoming refractory CIDP, we found a coefficient of 0.0411 (*p* = 0.020), suggesting a significant influence on being refractory CIDP. The ulnar nerve CMAP had a regression coefficient of-0.2963 (*p* = 0.056), suggesting a borderline significant influence on evolving into refractory CIDP. However, MRC sum score, median motor nerve distal latency and median motor nerve conduction velocity may not significantly affect the outcome, as summarized in Table 3.

**Table 3:** Multivariate logistic regression analyses for prognostic factors evolving to refractory CIDP.

Variable	Coefficient	Standard errors	*z*	*p*-value	95% CI
Duration	0.0411	0.018	2.328	**0.020**	(0.007, 0.076)
MRC	−0.0559	0.041	−1.366	0.172	(−0.136, 0.024)
ulnar_CMAP	−0.2963	0.155	−1.908	0.056	(−0.601, 0.008)
median_DML	−0.0637	0.060	−1.062	0.288	(−0.181, 0.054)
median_CV	−0.0018	0.030	−0.061	0.952	(−0.061, 0.057)

CI, confidence interval; CMAP, compound muscle action potential; CV, conduction velocity；DML, distal motor latency; MRC, Medical Research Council Sum Score.The value ‘0.020’ meaned *p* value was smaller than 0.05.

## Discussion

Refractory CIDP is a challenging subset of CIDP and a comprehensive understanding of its clinical profile remains to be further elucidated. Our study describes the clinical and electrophysiological features of patients with refractory CIDP. Compared to non-refractory CIDP patients, refractory CIDP patients present with more severe clinical neurological functional impairment and peripheral nerve damage demonstrated by electrophysiological studies. Additionally, disease duration can be considered as an independent prognostic risk factor for progressing to refractory CIDP. Importantly, four disease course patterns of refractory CIDP are described: a relapsing–remitting form, a stable form, a secondary progressive form and a primary progressive form.

The concept of refractory CIDP has been discussed for several years, but its definition remains inconsistent. In previous studies, three primary definitions have been discussed: (1) patients with poor treatment outcomes based on neurologists’ personal experiences and perspectives regarding treatment outcomes (6, 11), (2) patients with CIDP who do not respond to one of the three first-line therapies or are unable to continue these treatments due to adverse effects (10, 21, 22) or (3) patients with CIDP who do not respond to two of the three first-line or fail to respond to a combination of first-line and second-line therapies (8, 17, 18, 23, 24). To comprehensively describe the clinical profile of refractory CIDP, we adopt the third definition, which is more concise and objective. Moreover, to identify the specific characteristics of refractory CIDP, we excluded patients with autoimmune nodopathy, CISP, CCPD and monoclonal gammopathy related neuropathy from our study. Our findings showed that under the new background of the 2021 EAN/PNS guideline and our definition of refractory CIDP, 43.1% of CIDP patients presented as refractory CIDP, a significantly higher proportion compared to previously reported (3).

Refractory CIDP patients more often had a longer disease duration from symptom onset to diagnosis, namely diagnostic delay. In particular, longer disease duration has been demonstrated as an independent risk factor for CIDP patients transitioning into a refractory state. Diagnostic delay is a common issue in CIDP. Studies have shown that there is an average delay of 12 to 40 months between the onset of symptoms and diagnosis (7, 25). This delay often results in inappropriate treatment being administered too late. A delay in diagnosis can cause axonal injury to accumulate, which can lead to increased disability that may be irreversible even with treatment. Additionally, compared with non-refractory CIDP, refractory CIDP had more severe functional impairment at the inclusion entrance, as reflected by the lower MRC sum score. This could potentially be linked to a delay in diagnosis. Therefore, it is crucial to diagnose the condition quickly and start the treatment early to avoid irreversible disability.

In this research, electrophysiological studies provided further confirmation of a correlation between the severity of peripheral nerve impairment, characterized by more extensive demyelination and pronounced axonal loss, and the refractory nature of CIDP. It has also been established that axon loss is a significant long-term adverse prognostic factor in CIDP (7, 11), as evidenced by a greater decrease in CMAP demonstrated by nerve conduction study and the presence of axon loss in nerve biopsy specimens (6, 26, 27). Furthermore, our study has identified that severe demyelinating lesions serve as significant prognostic risk factors for adverse outcomes. It is widely acknowledged that demyelinating lesions could cause secondary axonal damage. As the disease progresses, if disease progression is not adequately controlled, such secondary damage may lead to irreversible axonal impairment.

This study aims to establish a more comprehensive foundation for precision treatment by identifying distinct disease course patterns within refractory CIDP. These include the relapsing–remitting, primary progressive, secondary progressive, and stable patterns. The relapsing–remitting form accounted for approximately half of the patients with refractory CIDP. The most striking characteristic in this group is that patients’ functional disability can fluctuate between normal and reduced levels, resembling the disease course pattern observed in relapsing–remitting multiple sclerosis (28). However, we observed that the level of disability during the last follow-up in the remission stage was more severe than that in the initial remission stage. This suggested that frequent relapses may lead to accumulating injuries, eventually resulting in irreversible impairment.

The stable group poses a significant challenge in clinical practice, as it becomes difficult to determine the true effectiveness of the ongoing treatment. Although it has a relatively stable condition, the effectiveness of current treatment or the possibility of responding to further attempted treatment could not be certainly identified. This uncertainty makes it challenging to decide whether to suspend the current treatment regimen and explore alternative therapies or to continue with the present medication until the desired effectiveness is observed.

Three patients presented with a primary progressive disease pattern and the diagnosis was carefully verified and confirmed. Previous studies have also reported that the progressive course pattern accounted for 6.7% of CIDP patients (6). Given the continued progression experienced by patients with a primary progressive or secondary progressive course, it is imperative that these individuals receive highly effective treatment in the early stage. This proactive approach is aimed at mitigating the potential for further axonal damage.

Our study has certain limitations. Firstly, the sample size was relatively small. As a retrospective study, there may be inherent biases in the clinical data. In HSPN database, patients who have a long-term and effective response might not have a regular follow-up and could be lost while patients with a poor treatment outcome have high compliance and might have a regular follow-up. And thus, the high proportion of refractory CIDP in our study may result from such a selective bias. Furthermore, it should also be noted that in China IVIg is limited availability and high cost, making it difficult for many patients to access or afford adequate treatment courses. Consequently, CIDP patients receiving IVIg as therapy often cannot afford to undergo a sufficient treatment course. This limitation often leads to rapid relapse and worsening of symptoms, contributing to the refractory nature of the disease. Additionally, this study did not explore the dynamic evolution of the clinical course and the associated conversion relationships. Nevertheless, it is important to note that this research provides insights and presents a relatively comprehensive clinical profile of refractory CIDP. It expands our understanding of the disease’s clinical manifestations within the context of the 2021 EAN/PNS guideline. Despite the limitations, our study provides a more accurate reflection of the refractory characteristics of CIDP.

## Conclusion

This study provided a comprehensive description of refractory CIDP, addressing its clinical features, classification of clinical course, electrophysiological characteristics, and prognostic factors, effectively elucidating its various aspects. These findings contribute to a better understanding of this challenging subset of CIDP and might be informative for management and treatment strategies.

## Data availability statement

The data analyzed in this study is subject to the following restrictions: the dataset includes data from our private patient database. Requests to access these datasets should be directed to Victor Zheng email: 19211220083@fudan.edu.cn.

## Ethics statement

The studies involving humans were approved by Ethics Committee of Huashan hospital, Fudan university. The studies were conducted in accordance with the local legislation and institutional requirements. The participants provided their written informed consent to participate in this study.

## Author contributions

YoZ: Conceptualization, Data curation, Formal analysis, Investigation, Methodology, Software, Validation, Visualization, Writing – original draft, Writing – review & editing. JH: Data curation, Formal analysis, Writing – original draft, Writing – review & editing. CS: Conceptualization, Investigation, Writing – review & editing. KQ: Data curation, Writing – review & editing. YaZ: Data curation, Writing – review & editing. BL: Writing – original draft. JS: Data curation, Writing – review & editing. JX: Validation, Writing – review & editing. SL: Resources, Writing – review & editing. JLu: Data curation, Methodology, Writing – review & editing. CZ: Data curation, Methodology, Validation, Writing – review & editing, Conceptualization, Supervision. JLi: Conceptualization, Data curation, Formal analysis, Investigation, Methodology, Supervision, Validation, Writing – review & editing.
