# Supplementary material for: Insights into refractory chronic inflammatory demyelinating polyneuropathy: a comprehensive real-world study
Source: Front Neurol. 2024 Jan 31;15:1326874. doi: 10.3389/fneur.2024.1326874 (PMC10865491; doi:10.3389/fneur.2024.1326874)
Supplement: Supplementary file 1 [file Table_1.DOCX]

Table S1 Comorbidities of CIDP

| Comorbidities | Refractory CIDP(n=25) | Non- refractory CIDP (n=33) | p-value |
| --- | --- | --- | --- |
| Type II diabetes | 2 | 3 | NS |
| Kidney disease | 0 | 2 | NS |
| Hypertension | 3 | 4 | NS |
| Primary aldosteronism | 0 | 1 | NS |
| Hepatitis B | 1 | 0 | NS |

Abbreviation: NS, no significance
