# Supplementary figures and images for: Insights into refractory chronic inflammatory demyelinating polyneuropathy: a comprehensive real-world study
Source: Front Neurol. 2024 Jan 31;15:1326874. doi: 10.3389/fneur.2024.1326874 (PMC10865491; doi:10.3389/fneur.2024.1326874)

Supplementary Figure 1

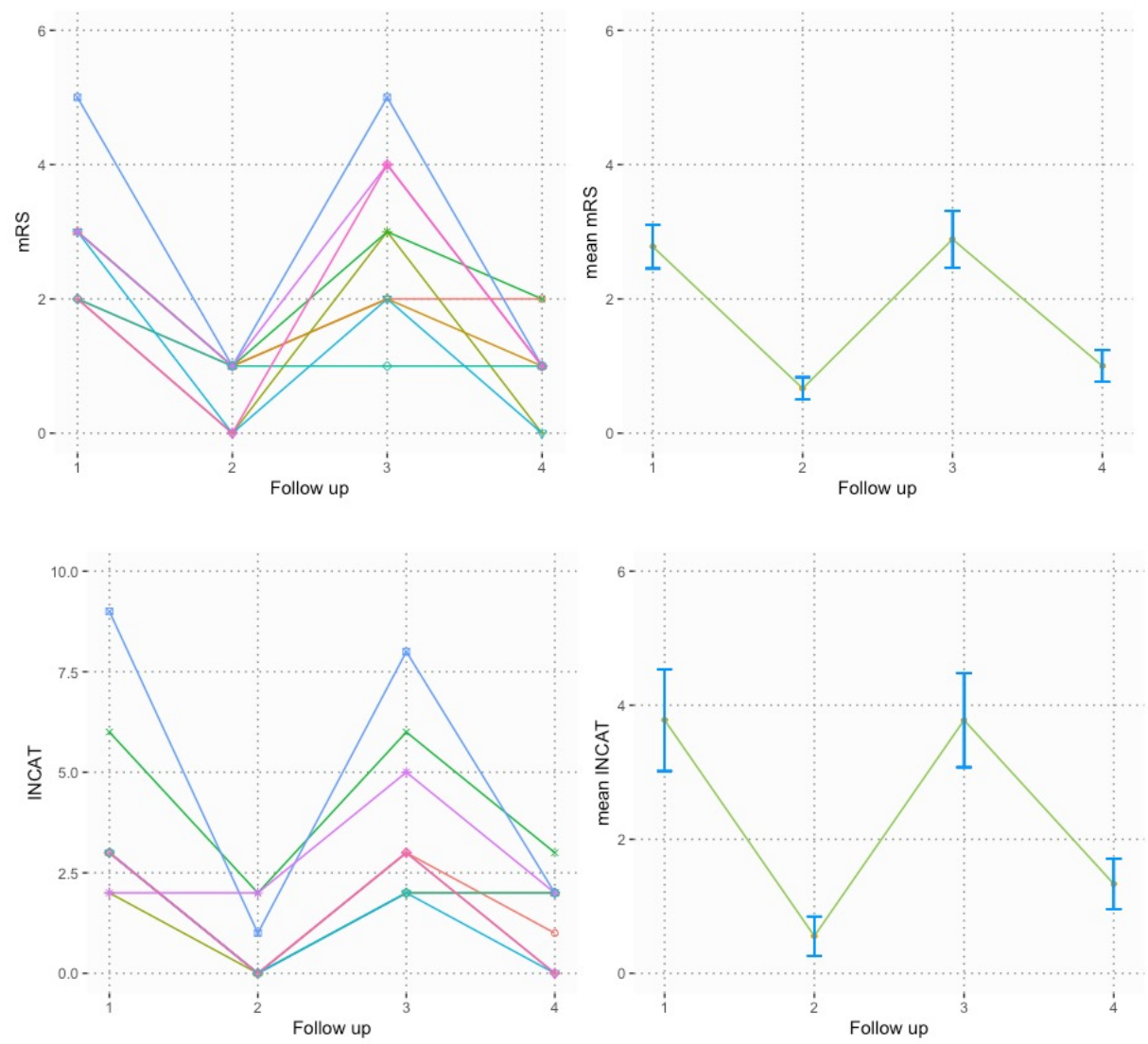

Supplementary Figure 2

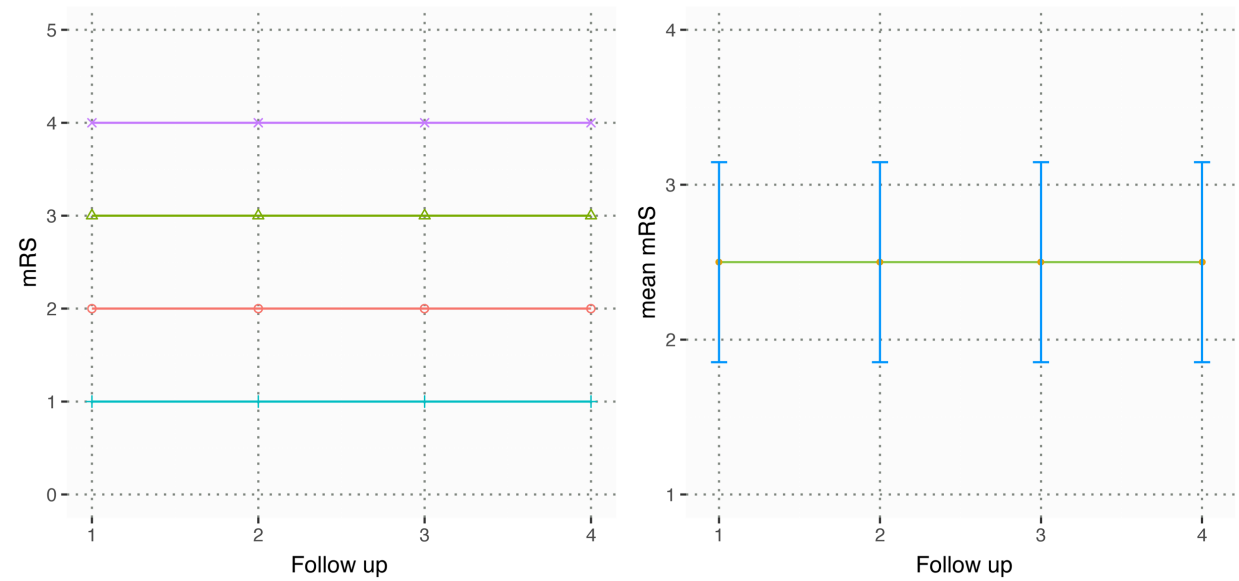

Supplementary Figure 3

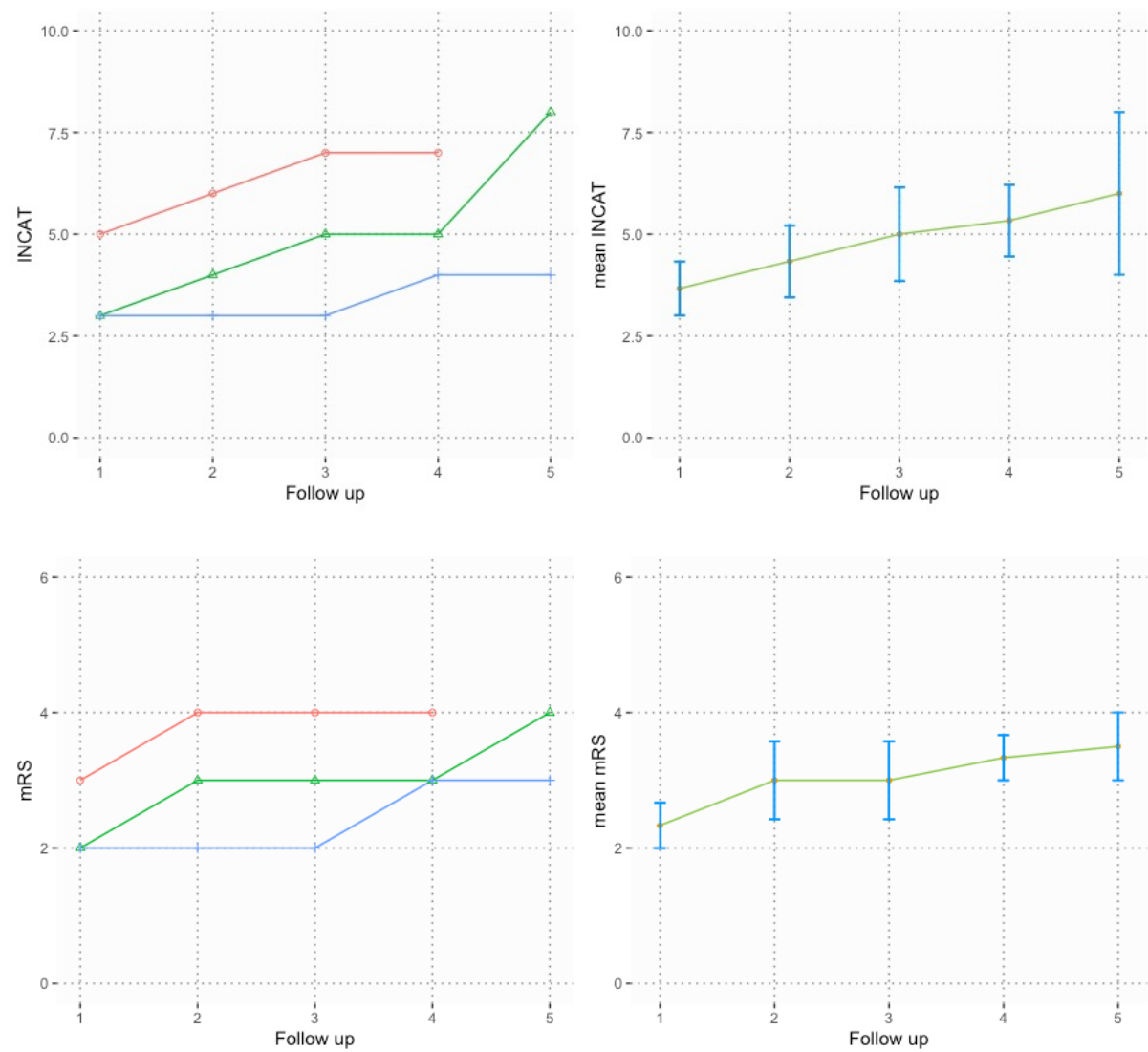

Supplementary Figure 4

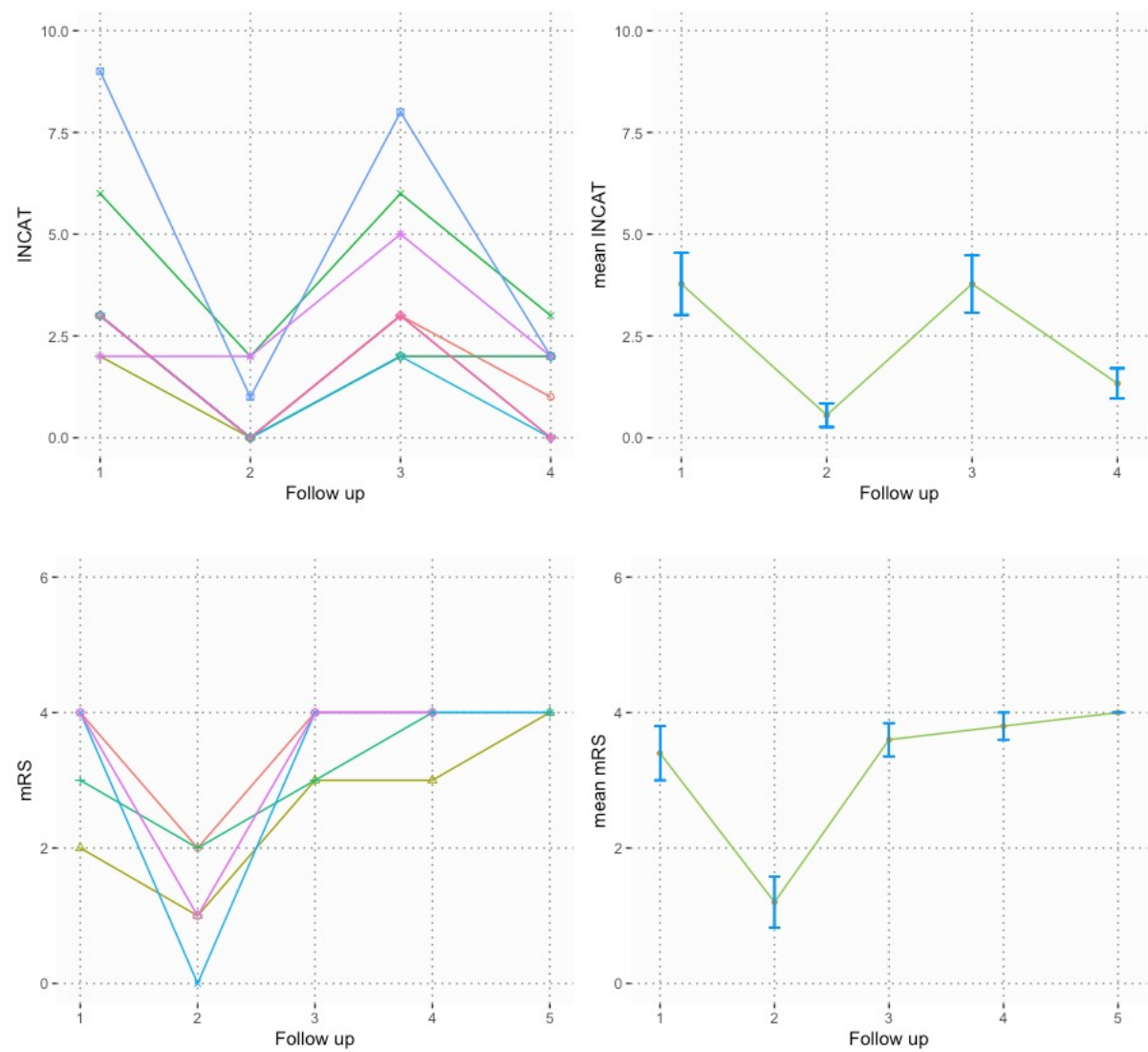

Supplement: Supplementary file 2 [file Data_Sheet_1.PDF]
